# Caloric Restriction Mimetics as Priming Agents of Mesenchymal Stem Cells Secretome to Enhance Regenerative Responses to Parkinson’s Disease

**DOI:** 10.3390/molecules30112260

**Published:** 2025-05-22

**Authors:** Bárbara Carneiro-Pereira, Filipa Ferreira-Antunes, Jonas Campos, António J. Salgado, Belém Sampaio-Marques

**Affiliations:** 1 Life and Health Sciences Research Institute (ICVS), School of Medicine, University of Minho, Campus de Gualtar, 4710-057 Braga, Portugal; pg52808@alunos.uminho.pt (B.C.-P.); id11400@alunos.uminho.pt (F.F.-A.); id9533@alunos.uminho.pt (J.C.); asalgado@med.uminho.pt (A.J.S.); 2 ICVS/3B’s—PT Government Associate Laboratory, 4805-017 Guimarães, Portugal

**Keywords:** Mesenchymal Stem Cells, secretome, priming, caloric restriction mimetics, neurodegeneration, Parkinson’s disease

## Abstract

Parkinson’s disease (PD) is a neurodegenerative disorder primarily defined by the deterioration of motor function and characterized by the loss of dopaminergic neurons in the nigrostriatal system. Although it is the second most prevalent disorder of the central nervous system, current treatments primarily focus on symptom management and modestly slowing disease progression, ultimately failing to preserve the long-term quality of life of a substantial proportion of affected individuals. Innovative therapies that can restore neuronal function have emerged, such as the use of the secretome of Mesenchymal Stem Cells (MSCs) due to their rich composition of bioactive molecules. This therapy exhibits robust paracrine activity that drives most of the self-renewal capacity, differentiation potential, and immune regulation of MSCs without presenting compatibility issues often associated with stem cell-based therapies. While conceptually appealing, the clinical application of this approach is still limited by the availability and proliferation capacity of MSCs, as it impacts not only secretome production but also its quality. Various protocols have been developed to enhance secretome action by adding various compounds to cell culture media, given the high environmental plasticity of MSCs. Some of the compounds already used are Caloric Restriction Mimetics (CRMs), molecules that mimic Caloric Restriction (CR) conditions, which have been demonstrated to extend lifespan and reduce age-related diseases in various organisms. While not sufficient to cure neurodegenerative disorders, these compounds may potentiate secretome efficiency by enhancing autophagy pathways and relieving oxidative stress burden from MSCs. Therefore, in this article, we aim to explore the effects of CRMs priming on MSCs and how it may help bridge existing gaps in regenerative therapies for PD.

## 1. Introduction

Parkinson’s disease (PD) is the second most common neurodegenerative disorder, affecting over 10 million people worldwide [1]. Pathologically, it is characterized by the presence of misfolded and aggregated α-synuclein (asyn), degeneration of dopaminergic neurons in the substantia nigra pars compacta (SNpc), and dopamine depletion in the striatum, leading to both non-motor and motor impairments [2]. The dopamine precursor amino acid l-3,4-dihydroxyphenylalanine (l-DOPA) was first introduced in 1950 as an approach to counteract striatal dopamine depletion and remains the “gold standard” symptomatic therapy for PD [3]. However, l-DOPA does not effectively halt or reverse neurodegeneration. Instead, it functions as a dopamine agonist, requiring continuous dose adjustment, and patients frequently develop drug resistance over time. Thus, disease-modifying treatments that halt the gradual degeneration of dopaminergic neurons remain unmet needs.

In this sense, Mesenchymal Stem Cells (MSCs) have emerged as a promising therapeutic approach owing to their unique features, such as self-renewal, differentiation capacities, and immunomodulatory properties [4]. Several studies have highlighted that the therapeutic potential of stem cells is mainly due to their paracrine effect through the secretion of soluble factors and extracellular vesicles (EVs), collectively known as their secretome [5]. In recent years, several studies have shown promising results for MSC-secretome treatment in in vitro and in vivo models [6,7,8,9,10,11,12,13,14,15]. Despite these promising results, further advancements are needed to enhance therapeutic efficacy and improve outcomes. Modulating its composition presents a promising strategy for targeting specific dysregulated pathways or shifting its profile toward a more regenerative state.

Caloric restriction (CR) is a well-established intervention that extends lifespan and delays age-related diseases by modulating longevity-relevant pathways like adenosine-monophosphate-activated protein kinase (AMPK), mechanistic target of rapamycin (mTOR), sirtuin-1 (SIRT1), and forkhead box O (FOXO). However, its strict adherence is challenging, prompting interest in CR mimetics (CRMs) and bioactive molecules, such as quercetin, spermidine, resveratrol, and metformin, which replicate CR’s anti-aging effects without dietary limitations [16,17,18]. Their potential to modulate oxidative stress, autophagy, and mitochondrial function opens new therapeutic avenues for neurodegenerative diseases like PD, particularly when combined with the regenerative and immunomodulatory secretome of MSCs.

In this chapter, we provide a concise overview of the key impaired pathways in PD and explore the potential of the MSC-derived secretome as a therapeutic strategy. Additionally, we discuss innovative approaches to optimize the production of MSC-derived secretome and methods to modulate their secretory profiles to address specific therapeutic needs. Our focus will be on anti-aging strategies, namely CRMs, and their potential to enhance the regenerative capacity of this therapeutic approach.

## 2. Parkinson’s Disease

PD was first described by James Parkinson in 1817 as a shaking palsy characterized by “involuntary tremulous motion, with lessened muscular power, in parts not in action and even when supported; with a propensity to bend the trunk forwards, and to pass from a walking to a running pace: the senses and intellects being uninjured” [19,20]. Over two centuries have passed, and massive scientific breakthroughs have been achieved at the clinical and experimental levels, revealing the true complexity of PD, which is now described as a multifactorial disease. The neuropathological criteria for PD diagnosis are moderate to severe neuronal loss in the SNpc associated with widespread Lewy pathology, which is characterized by the presence of asyn immunoreactive neuronal inclusions [21]. Moreover, additional neuronal loss assessment may include the presence of fibrillary astrocytosis and extraneuronal neuromelanin [22].

### 2.1. Pathophysiology

PD is a clinicopathological syndrome whose major cardinal motor features are progressive asymmetric slowness of movement (bradykinesia), rigidity, tremor, and gait disturbances. The pathological feature that correlates with clinical PD symptoms is neuronal loss in the basal ganglia, an interconnected group of subcortical and brainstem nuclei that mainly control the initiation and execution of movements. Specifically, the loss of dopaminergic neurons in the midbrain SNpc and denervation of the striatum compromise neuronal signaling in this pathway, resulting in the aforementioned motor symptoms [23]. Degeneration of dopaminergic (DA) neurons is reported to usually begin in the lateral ventral tier of the SNpc, containing the neurons that project to the dorsal putamen of the striatum, and is estimated to represent a 68% cell loss at the onset of motor symptoms when PD diagnosis is performed [24]. Nonetheless, in the early stages of PD, striatal dopaminergic loss exceeds SN neuron cell death, suggesting retrograde degeneration of the nigrostriatal pathway [25,26].

Another neuropathological hallmark of PD is the presence of aberrant aggregates, primarily composed of asyn, known as Lewy pathology [27]. aSyn is a 140-amino-acid presynaptic neuronal protein abundantly found in the brain that is both genetically and neuropathologically linked to PD [28]. This protein can remodel itself within the plasma membrane, adopting an alpha-helical conformation. However, it can also misfold into a structure rich in cross-beta sheets, leading to abnormal phosphorylated protein aggregation in the form of Lewy bodies (LBs). LBs are present in both neuronal cell bodies and dystrophic axonal neurites (Lewy neurites) [29,30]. Postmortem studies have proposed that LB pathology correlates with disease progression in PD and is therefore considered a marker of disease progression. Besides being a disease hallmark, increasing evidence has shown that asyn aggregates can contribute to neuronal dysfunction and death [31].

### 2.2. Cellular and Molecular Mechanisms

**Figure 1:**
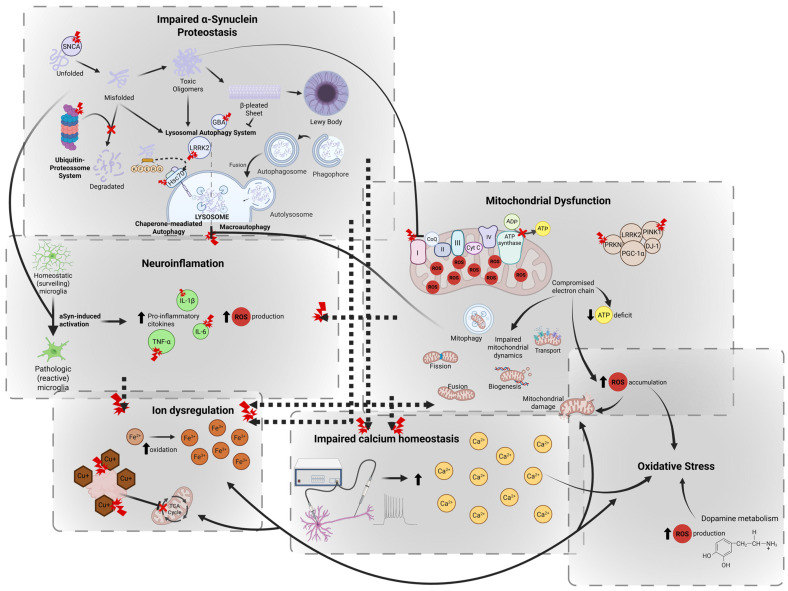
Molecular pathways of Parkinson’s disease (PD).This figure illustrates the complex and interrelated molecular mechanisms underlying PD, with asyn dysfunction at the core. Misfolding and aggregation of asyn result in toxic oligomers and β-sheet fibrils that accumulate as Lewy bodies. Under normal conditions, asyn is cleared via the ubiquitin-proteasome system and lysosomal degradation, including chaperone-mediated autophagy. Mutations in genes such as *LRRK2*, *GBA*, and *VPS35* impair proteostatic pathways, promoting intracellular asyn accumulation. These aggregates activate microglia, shifting them from a homeostatic to a reactive state and triggering the release of pro-inflammatory cytokines (e.g., TNF-α, IL-1β, and IL-6), thereby initiating chronic neuroinflammation. Inflammatory mediators enhance ROS production, thereby exacerbating cellular stress. Mitochondrial dysfunction—marked by impaired electron transport, reduced ATP production, and increased ROS—further contributes to PD pathogenesis. Disrupted mitochondrial dynamics (fission, fusion, mitophagy, and biogenesis) and mutations in the genes that encode PINK1, PRKN, LRRK2, DJ-1, and PGC-1α exacerbate mitochondrial failure, creating a vicious cycle of energy depletion and oxidative stress. Metal ion dyshomeostasis, particularly involving iron (Fe^2+^ to Fe^3+^) and copper (Cu^+^), intensifies oxidative damage. Iron accumulation accelerates ROS generation via Fenton chemistry, while copper imbalance disrupts redox homeostasis. Both also facilitate asyn aggregation. Calcium (Ca^2+^) dysregulation further aggravates mitochondrial stress and excitotoxicity and impairs autophagic clearance, while elevated intracellular Ca^2+^ promotes α-syn aggregation. Oxidative stress emerges as a central consequence and amplifier of these pathologies, driven by mitochondrial ROS, dopamine metabolism, inflammation, and metal ion imbalances. Cumulative oxidative damage to proteins, lipids, and DNA perpetuates dysfunction in proteostasis and mitochondrial integrity, reinforcing a self-sustaining degenerative loop. Together, these processes converge to drive the progressive loss of dopaminergic neurons, a characteristic of PD. PD—Parkinson’s disease; aSyn-α-synuclein; LRRK2—leucine-rich repeat kinase 2; *GBA*—glucosylceramidase beta; VPS35—vacuolar protein sorting 35; TNF-α—tumor necrosis factor α; IL—interleukin; ROS—reactive oxygen species; PINK1—phosphatase and tensin homolog induced novel kinase 1; PRKN—Parkin; PGC-1α—peroxisome proliferator-activated receptor-γ coactivator 1-α. Created in Bio Render (https://www.biorender.com/, accessed on 8 April 2025).

The complex pathophysiology of PD seems to be a result of the interplay of the dysfunction of several pathways that culminate in neurodegeneration [2,32,33]. In this section, we explore the most studied cellular and molecular mechanisms associated with PD pathophysiology (Figure 1).

#### 2.2.1. Impaired α-Synuclein Proteostasis

Over the last few decades, several mutations (e.g., synuclein alpha (*SNCA*), leucine-rich repeat kinase 2 (*LRRK2*), glucosylceramidase beta (*GBA*), phosphatase and tensin homolog induced novel kinase 1 (*PINK1*), and *DJ-1*) have been identified as possible triggers for aggregation, being correlated with asyn imbalance, asyn overproduction, or increasing the likelihood of misfolding and oligomerization. However, an imbalance between protein synthesis and degradation may also arise from alterations in the molecular pathways responsible for the clearance of misfolded proteins, either related to aging or genetic mutations [34]. In addition to asyn, LBs contain other proteins, including ubiquitin, tau, parkin, oxidized/nitrated proteins, cytoskeletal proteins, heat shock proteins, and proteasomal and lysosomal elements. This further supports the notion that proteostatic mechanisms are severely impaired in PD, potentially serving as major contributors to disease progression. Thus, impaired asyn proteostasis—or protein homeostasis—is being widely studied to understand PD’s progression and to unveil new therapeutic strategies.

The ubiquitin-proteasome system (UPS) and lysosomal autophagy system (LAS) are the two major intracellular proteolytic systems involved in asyn degradation. The UPS is thought to be mainly responsible for the degradation of short-lived soluble proteins [35], while under pathologic conditions, LAS is suggested to be responsible for the vesicle-mediated degradation of long-lived proteins. This can happen via macroautophagy (hereafter called autophagy) or chaperone-mediated autophagy (CMA). Autophagy is a tightly regulated process involving the formation of double-membrane-bound structures (autophagosomes) to engulf intracellular constituents, thereby generating autophagic vacuoles that subsequently fuse with lysosomes for degradation, creating autophagolysosomes [36,37]. CMA is also responsible for lysosomal degradation, although only of a very specific subset of soluble cytosolic proteins [36]. aSyn belongs to this selective group, as it contains a KFERQ-like motif that is recognized by the cytosolic chaperone heat shock cognate protein 70 (HSC70) [37,38]. Supporting evidence for the involvement of impaired proteostasis in PD pathogenesis comes from the observation of increased expression of autophagosomes, decreased expression of lysosomal marker proteins, and proteins of chaperone-mediated autophagy (lysosomal-associated membrane protein 2A (LAMP2A) and HSC70) in postmortem analysis of PD patients’ brains [39,40,41].

#### 2.2.2. Mitochondrial Dysfunction

Mitochondria are essential for maintaining neuronal function through fission, fusion, transport, autophagic degradation (mitophagy), and biogenesis. Even subtle disruptions in these mechanisms can have profound consequences on cellular health and significantly influence disease progression [42]. The first indications of mitochondrial involvement in PD progression arose from studies on brain tissue samples from patients with PD, which revealed deficits in the activity of mitochondrial complex I, a key component of the electron transport chain [43,44]. This energy deficiency is potentially an upstream and early neurodegenerative event in PD and has been associated with axonal degeneration [2]. Complex I inhibitors, such as 1-methyl-4-phenylpyridinium ion (MPP+) and rotenone, induce irreversible lesions in dopaminergic neurons when systemically administered to animal models and have been largely used to develop animal models of PD [45,46]. Furthermore, asyn accumulation inside mitochondria has been proposed to play a role in evoking mitochondrial complex I deficits [47,48].

#### 2.2.3. Oxidative Stress

A key factor contributing to the vulnerability of dopaminergic neurons is their high energy demand, which is closely linked to increased reactive oxygen species (ROS) production and accumulation. Oxidative stress occurs when there is an imbalance between the production of ROS and cellular antioxidant activity [49]. This imbalance is thought to be the main cause of cell death in both idiopathic and genetic cases of PD [50]. Indeed, the brain tissue of patients with PD has shown increased levels of oxidized lipids, proteins, and DNA [51,52]. However, it is not clear whether it is a cause or consequence of other cellular dysfunctions, such as mitochondrial dysfunction, impaired calcium homeostasis, neuroinflammation, and iron accumulation [49]. In fact, dopamine production is a major source of ROS production, which may also explain the vulnerability of dopaminergic neurons to neurodegeneration [53]. The imbalance leading to oxidative stress may also arise from decreased activity of antioxidant proteins, such as DJ-1, which is linked to autosomal recessive, early-onset PD [54,55].

#### 2.2.4. Impaired Calcium Homeostasis

Alterations in calcium homeostasis are particularly detrimental to dopaminergic neurons, especially those in the substantia nigra, due to their reliance on calcium-dependent pacemaking activity to maintain spontaneous firing and neurotransmitter release [36,56]. These neurons use L-type voltage-gated calcium channels (particularly Ca_v_1.3) to generate rhythmic electrical activity, which makes them uniquely dependent on tightly regulated calcium influx [56,57].

However, this constant calcium entry imposes a high metabolic demand and exposes neurons to chronic calcium stress. Unlike many other neuron types, dopaminergic neurons have relatively low levels of calcium-binding proteins, such as calbindin, which limits their capacity to buffer intracellular calcium effectively. This makes them more vulnerable to fluctuations in calcium levels [58].

Disruption of calcium homeostasis in PD can occur not only as a primary vulnerability but also as a secondary effect of disease-related processes. For example, asyn aggregation can disrupt calcium channels and signaling pathways by shifting calcium pump activation from the plasma membrane Ca^2+^ ATPase (PMCA) to the sarcoplasmic/endoplasmic reticulum Ca^2+^-ATPase (SERCA), leading to intracellular calcium overload [59]. Moreover, mitochondrial dysfunction, which is common in PD, impairs calcium uptake and buffering, while ER stress can disturb calcium storage and release [56,60]. Together, these factors contribute to intracellular calcium overload, oxidative stress, and the activation of calcium-dependent enzymes that promote neurodegeneration [36,56,60].

#### 2.2.5. Ion Dysregulation

Several neuronal functions, including metabolism, neurotransmission, and myelination, depend on iron levels [61]. Under normal physiological conditions, excess iron is sequestered in ferritin and neuromelanin, limiting the availability of redox-active (free) iron [61,62]. The increased iron accumulation observed in the SNpc of patients with PD highlights disrupted iron metabolism as a key contributor to neurodegeneration [49,62,63]. However, whether elevated iron levels drive neurodegeneration or arise as a consequence of oxidative stress, inflammation, excitotoxicity, mitochondrial dysfunction, and impaired proteostasis remains unclear [61].

Iron-mediated cellular damage primarily results from oxidative stress. We hypothesized that intracellular iron overload, in combination with hydrogen peroxide generated during normal metabolism, leads to the formation of highly toxic hydroxyl radicals. These radicals trigger cellular damage, lipid peroxidation, and ultimately apoptosis [61,62].

Beyond classical oxidative injury, recent insights have identified ferroptosis, an iron-dependent form of regulated cell death, as a key mechanism in PD pathology. Ferroptosis is characterized by the accumulation of lipid peroxides and reactive oxygen species, with neuronal susceptibility heightened by impaired glutathione peroxidase 4 (GPX4) activity and disrupted iron handling [64,65]. Elevated markers of lipid peroxidation and altered expression of iron transport and storage proteins, such as divalent metal transporter 1 (DMT1) and ferritin, have been observed in the brains of patients with PD, supporting the involvement of ferroptosis [65,66]. Notably, pharmacological inhibition of ferroptosis (e.g., via acteoside) has shown neuroprotective effects in PD models by restoring glutathione levels and reducing lipid peroxidation, suggesting its potential therapeutic value [66].

In parallel, a newly described form of metal-dependent cell death, cuprotosis, has emerged as relevant to neurodegeneration. Cuprotosis is mediated by copper binding to lipoylated mitochondrial proteins in the tricarboxylic acid (TCA) cycle, leading to proteotoxic stress, metabolic dysfunction, and cell death [67]. Although the role of copper in PD remains less defined than that of iron, evidence of altered copper homeostasis (e.g., dysregulated ATP7A/ATP7B transporters) and mitochondrial dysfunction in PD neurons suggests a possible contribution [68,69]. Dysregulation of copper transporters, such as ATP7A and ATP7B-critical for maintaining brain copper balance, may further sensitize neurons to cuprotosis, highlighting the need to explore this pathway in future PD research [68,69].

#### 2.2.6. Neuroinflammation

Neuronal loss in PD has also been linked to chronic neuroinflammation, primarily driven by microglia, which are the resident innate immune cells of the central nervous system [70]. The role of neuroinflammation in neurodegeneration is supported by postmortem analyses, as well as genetic and imaging studies [71]. Microglia play a crucial role in clearing neuronal debris following injury or toxic insults [50]. Interestingly, several lines of evidence suggest that activated microglial cells directly engulf asyn in an attempt to clear it from the extracellular space, either as a result of apoptotic neuron death or from mechanisms of cellular release [72,73,74,75]. However, while microglial activation for cellular damage resolution is a key player in brain homeostasis, chronic asyn-induced activation leads to a sustained pro-inflammatory state, marked by the release of neurotoxic factors such as ROS, nitric oxide (NO), and a cascade of pro-inflammatory cytokines, including interleukin-1β (IL-1β), IL-6, and tumor necrosis factor-alpha (TNF-α) [50,70,76]. This cytokine surge is tightly linked to the activation of transcriptional regulators such as nuclear factor κ B (NF-κB) and inflammasome complexes like NLRP3, both of which have been implicated in PD pathogenesis [77]. Chemokines, such as monocyte chemoattractant protein-1 (MCP-1) and CX3CL1 (fractalkine), further contribute to the recruitment and activation of peripheral immune cells, especially when the blood–brain barrier (BBB) is compromised, a common feature in PD [78]. Concurrently, adhesion molecules, including ICAM-1 and VCAM-1, are upregulated in cerebral endothelial cells, promoting leukocyte infiltration and amplifying the neuroinflammatory response [78]. These molecular and cellular alterations not only sustain microgliosis but also activate astrocytes (astrogliosis), which further propagate an inflammatory milieu [79].

Alterations in genes commonly associated with familial PD—such as SNCA, LRRK2, VPS35, Parkin (PRKN), PINK1, DJ-1, and GBA—have been shown to modulate immune signaling and mitochondrial integrity. For instance, mutated asyn interacts with neuromelanin and mitochondrial membranes, triggering NF-κB signaling and the release of IL-1β, IL-6, and TNF-α, thus contributing to oxidative stress and promoting the aggregation of misfolded asyn into Lewy bodies. These processes impair mitochondrial quality control mechanisms, such as mitophagy and autophagy, accelerating dopaminergic neurodegeneration [79].

Animal models of PD have corroborated this interplay, demonstrating how neuroinflammation interacts synergistically with mitochondrial dysfunction and proteinopathy to drive disease progression. Moreover, recent evidence suggests an immunomodulatory role for dopamine itself in the regulation of inflammatory responses. The inherent vulnerability of dopaminergic neurons in the substantia nigra may also be exacerbated by inflammatory stress due to their unique metabolic demands, high iron content, specific calcium channel expression, and low intrinsic antioxidant defenses [80].

Although the precise role of immunity in the etiology of PD remains under investigation, there is a consensus that maladaptive immune responses contribute significantly to disease progression. Initial activation of innate immune mechanisms may serve protective roles; however, chronic dysregulation leads to persistent inflammation, cellular dysfunction, and irreversible neurodegeneration.

### 2.3. Current Treatment Limitations

Despite significant advances in understanding the pathophysiology of PD, effective disease-modifying therapies remain unavailable. Current treatments are purely symptomatic and aim to alleviate both motor and non-motor symptoms. While dopamine-based therapies effectively manage early motor symptoms, non-dopaminergic approaches are often required to treat non-motor manifestations. Pharmacological treatment is typically complemented by non-pharmacological interventions, including rehabilitative therapies (physical, occupational, and speech therapy) and regular physical exercise. Palliative care also plays a crucial role in disease management [81].

More advanced and invasive treatments, such as deep brain stimulation (DBS) and pump therapies, are generally reserved for patients in advanced stages or those experiencing complications, such as motor fluctuations, medication-resistant tremors, or dyskinesias [81,82]. However, these interventions are significantly more expensive than standard pharmacological treatments and also have a history of loss of efficacy over time [83].

Thus, the development of disease-modifying therapies that halt dopaminergic neurodegeneration and target multiple pathogenic pathways involved remains an urgent unmet need. In this sense, the use of MSCs has emerged in the last few decades as a promising therapeutic approach for a variety of neurodegenerative disorders, including PD.

In parallel, gene therapy has gained popularity as an innovative and potentially transformative strategy for PD management. Early trials faced major safety concerns, including systemic inflammatory reactions and insertional mutagenesis. However, rapid technological advances have improved vector design, delivery methods, and safety profiles. Several gene therapy approaches have entered clinical trials, most notably those aiming to restore dopamine biosynthesis through the viral vector-mediated delivery of enzymes such as aromatic l-amino acid decarboxylase [84,85]. These therapies have shown motor improvements and reduced levodopa requirements, although challenges remain regarding surgical delivery and side effects like dyskinesia. Other gene therapy efforts have focused on delivering neurotrophic factors or enhancing GABAergic tone via glutamic acid decarboxylase (GAD) expression in the subthalamic nucleus, offering symptomatic relief without directly increasing dopamine levels. More recently, CRISPR/Cas9-based gene editing has emerged as a highly specific approach with the potential to correct pathogenic mutations or regulate asyn expression, although its clinical application in PD is still in the preclinical stages [85,86]. Despite promising results, the current limitations of gene therapy include the need for invasive neurosurgical procedures, lack of dose flexibility, high cost, and uncertainty of long-term efficacy compared to established interventions like DBS. Novel non-viral delivery systems, including nanoparticles and hydrogels, are being explored to address these challenges and enhance their clinical translation [85].

Together, these emerging strategies, ranging from regenerative cell-based therapies to targeted genetic modulation, represent the next frontier in the search for disease-modifying treatments for PD.

## 3. Mesenchymal Stem Cells

Cell-based therapy has long been proposed as an attractive strategy to replace degenerating dopaminergic neurons and thus restore the normal physiological pattern of striatal dopamine transmission in PD [4,87]. Mesenchymal stem cells (MSCs) are a heterogeneous group of multipotent, non-hematopoietic progenitor cells of mesodermal origin, characterized by their ability to self-renew, proliferate, and differentiate into various mesodermal lineages, such as osteoblasts, chondrocytes, and adipocytes. With the growing interest in cell-based therapies, MSCs have emerged as a top candidate cell source for several reasons. First, MSCs have widespread availability in the human body, and they can be isolated from the bone marrow, adipose tissue, umbilical cord, dental pulp, peripheral blood, and neonatal tissues, among others. Recently, protocols for deriving MSCs from induced pluripotent stem cells (iPSCs) have revolutionized the field of regenerative medicine [88]. MSCs are easy to isolate and expand in vitro and can be stored for a long time since they can maintain their viability and regenerative ability after cryopreservation. Additionally, their self-renewal and differentiation capacities, together with their low tumorigenic and immunogenic properties, which allow allogeneic transplantation approaches, make these cells very attractive for neurodegenerative disease therapies [89,90].

Although MSCs were initially proposed as tools for neuronal replacement due to their multipotency and migratory capabilities, recent findings have significantly shifted this paradigm. The relevance of MSCs differentiation into neuronal lineages is increasingly questioned for central nervous system (CNS) applications. Numerous studies have shown that while MSCs can adopt neuron-like morphologies and express neuronal genes and proteins in vitro, these changes are largely artifacts of the artificial culture environment rather than evidence of true neuronal differentiation [91,92,93,94,95]. Crucially, the acquisition of a mature neuronal phenotype, particularly the expression of functional depolarization-inducing voltage-gated sodium channels, has not been convincingly demonstrated [96,97]. In vivo studies purportedly showing MSC differentiation into neurons or glia often rely on early-stage markers such as nestin, βIII-tubulin, or GFAP, which MSCs can express even prior to induction [98,99]. Furthermore, experimental models have revealed that MSCs tend to fuse with resident neural cells rather than differentiate into them, and such fusion events are rare (<2%) and insufficient to account for the observed therapeutic outcomes [100,101]. Collectively, these findings support a growing consensus that, particularly in the context of CNS regeneration, the therapeutic benefits of MSCs arise not from direct cell replacement but from their paracrine activity—the so-called secretome—which modulates the local microenvironment, influences immune responses, and promotes endogenous repair processes [102].

### 3.1. Mesenchymal Stem Cells Secretome

MSCs secrete a variety of signaling molecules, including extracellular vesicles containing micro-RNAs (miRNAs) (e.g., miRNA-106b, miRNA-124a, and miRNA-181a-2-3p) and soluble proteins [growth factors (e.g., vascular endothelial growth factor (VEGF), brain-derived neurotrophic factor (BDNF), fibroblast growth factor 9 (FGF-9), epidermal growth factor (EGF), hepatocyte growth factor (HGF), leukemia inhibitory factor (LIF), neurotrophin-3 (NT-3), and transforming growth factor beta 2 (TGF-β2)), cytokines (e.g., IL-6, IL-8), chemokines (e.g., MCP-1, chemokine ligand 4 (CCL4), regulated upon activation, normal T cell expressed and secreted (RANTES) and interferon γ-induced protein 10 kDa (IP-10)) and other proteins (e.g., tissue inhibitor of metalloproteinase (TIMP-1) and osteoprotegerin (OPG))]. Several studies have shown the capacity of MSCs secretome to modulate several biological mechanisms under pathological environments, including promotion of neuroprotective mechanisms, inhibition of cell apoptosis, modulation of the immune system in order to counteract exacerbated inflammation processes, promotion of angiogenesis, induction of cell genesis and proliferation, promotion of stem cell migration to injured tissues, stimulation of re-epithelialization and extracellular matrix (ECM) remodeling mechanisms, as well as promotion of anti-fibrotic effects. The modulation of these processes has been reported to act synergistically in order to regenerate and recover from several human diseases, including PD [6,7,102,103,104,105,106,107].

### 3.2. Mesenchymal Stem Cells’ Therapeutic Effects in Parkinson’s Disease

Given the complex pathophysiology of PD, therapeutic approaches must target multiple mechanisms and pathways to ultimately promote neuroprotection and restore functionality. MSCs secretome has shown promising results in pathways of interest in PD.

#### 3.2.1. Neuroprotection

MSCs secretome has been shown to protect and reduce cellular loss in the nigrostriatal pathway following 6-OHDA-induced neuronal death in mouse and rat models [6,7,8,10,11,13,108,109,110,111,112]. These results have been attributed to several molecules with proposed roles in neuroprotection present in the secretome. In fact, proteomic analysis of human bone marrow-derived MSCs (hBM-MSCs) secretome revealed the presence of important neurotrophic factors, such as VEGF, BDNF, IL-6, and glial-derived neurotrophic factor (GDNF), as well as potential neuroregulatory molecules, namely DJ-1, cystatin C (CST3), glial-derived nexin, galectin-1, and pigment epithelium-derived factor [6,7].

Additionally, the MSC-derived secretome has been shown to promote axonal outgrowth and enhance neuronal connectivity within the CNS. This effect is primarily mediated by BDNF, which facilitates neurite extension and synaptic remodeling, indicating its potential role in functional recovery [113]. Furthermore, MSC-derived EVs containing specific miRNAs and proteins have been implicated in modulating signaling pathways critical for neuronal survival and neurogenesis [114,115]. Notably, MSC-derived EVs have been shown to preserve calcium homeostasis and prevent neuronal death by activating the phosphoinositide 3-kinase (PI3K)/ protein kinase B (Akt) signaling pathway, further supporting their therapeutic potential in PD [116].

#### 3.2.2. Clearance of α-Synuclein Aggregates

Given the importance of asyn aggregation in the pathology of PD, one of the main focuses of therapeutic approaches is their ability to induce clearance or reduce aggregate burden. In animal models of asyn aggregation, the secretome of MSCs can degrade extracellular asyn [13]. This effect is proposed to be partially mediated by metalloproteinase-2 (MMP-2) [117], and blocks clathrin-mediated endocytosis, thereby inhibiting asyn transmission [118]. Furthermore, several molecules with a proposed role against asyn aggregation or involved in proteostatic mechanisms have also been identified in the MSCs secretome (e.g., s BDNF, cofilin 1 (CFL1), heat shock protein family A member 8 (HSPA8), CST3, clusterin (CLU), VEGF-B, insulin-like growth factor (IGF1), ubiquitin C-terminal hydrolase L1 (UCHL1), and galectin (LGALS)). MSCs have been shown to modulate autophagy-lysosomal function and enhance asyn clearance in PD models [119]. In both in vitro and in vivo studies, MSCs’ paracrine activity significantly augmented autophagolysosome formation and attenuated asyn expression, which may have increased the survival of dopaminergic neurons against environmental neurotoxins. Specifically, the neuroprotective effect of MSCs is largely dependent on lysosomal activity mediated by autophagolysosome formation. The induction of autophagy is proposed to be related to the upregulation of Beclin-1 (BCEN1), an important positive regulator of mammalian autophagy. In addition, MSCs’ secretome has been shown to contain multiple factors involved in autophagy signaling. Notably, it influences the PI3K/Akt pathway and regulates various downstream targets that promote nutrient uptake, metabolism, cell growth, and proliferation [120,121]. This regulation includes the induction of autophagy-related genes, such as autophagy-related 12 (*ATG12*), *BCEN1*, and GABA type A receptor-associated protein like 1 (*GABARAPL1*) [122,123]. Therefore, the ability of the MSC-derived secretome to directly or indirectly modulate autophagy presents a promising therapeutic strategy for PD, potentially aiding in the regulation and prevention of asyn accumulation [120,121].

#### 3.2.3. Immunomodulation

As described previously, microglial activation and reactivity seem to play an important role in the development of PD pathophysiology. In animal models, modulating the inflammatory response has been explored as a target for therapeutic approaches [124]. Both pro- and anti-inflammatory cytokines have been identified in MSC secretomes. Some studies have shown that the anti-inflammatory action of the secretome is able to suppress microglia activation, potentially through paracrine modulation of the peripheral immune system. Key mediators of this effect include IL-6, IL-10, prostaglandin E2, and inducible indoleamine 2,3-dioxygenase (IDO) [125]. However, in other studies, MSCs’ secretomes have been shown to recruit microglia to the lesion site and induce phagocytosis [13]. Still, the specific mechanisms are unclear, and there is the possibility that the presence of both pro- and anti-inflammatory cytokines may have opposite effects. Therefore, it is essential to modulate or enrich certain molecules in the secretome that can be more favorable in specific cases.

#### 3.2.4. Mitochondrial Transfer and Bioenergetic Support

Recent studies have challenged the traditional concept of mitochondrial inheritance by demonstrating the horizontal transfer of mitochondria between mammalian cells [6]. This intracellular mitochondrial transfer is mediated by various structures, including tunneling nanotubes (TNTs) [126], EVs [127], gap junctions [128], and cell fusion mechanisms [129]. Importantly, healthy MSCs can release mitochondria within EVs (MitoEVs), which promote anti-inflammatory effects and restore energy metabolism in target cells [130]. This restoration of mitochondrial function helps rescue cells from apoptosis and restores their functions [131].

A growing body of research has highlighted the important effects of mitochondrial transfer in both in vivo and in vitro models of disorders associated with mitochondrial dysfunction [132]. For instance, a recent study demonstrated that mitochondria, both in their unmodified state (Mito) and conjugated with Pep-1 (P-Mito), were delivered intranasally to rats with 6-OHDA-induced lesions, a common animal model of PD. The intranasal delivery of these mitochondria improved the rotational and locomotor behaviors of lesioned animals compared to those of the control group. Additionally, increased survival of DA neurons was observed in lesions of the SN and striatum in Mito and P-Mito rats. This improvement was attributed to the restoration of mitochondrial function and reduction in oxidative damage in the lesioned SN [133].

#### 3.2.5. Blood−Brain Barrier Modulation

The pathophysiology of PD is closely linked to the disruption of the BBB, a critical interface regulating cerebral homeostasis. Emerging evidence suggests that the MSC-derived secretome plays a key role in preserving BBB integrity in PD. Recent studies have demonstrated that the MSCs’ secretome exerts protective effects against BBB damage induced by toxic asyn aggregates. Specifically, it has been shown that the secretome can mitigate the deleterious impact of these aggregates on the BBB, thereby preserving its structural and functional integrity [134]. Furthermore, MSCs exhibit neuroprotective and immunomodulatory properties in PD models. Therefore, MSCs and their secretome contribute to BBB modulation by regulating cell transporters, remodeling the extracellular matrix, and stabilizing cell junction components, which are vital for maintaining BBB integrity. Such alterations contribute to the restoration of the BBB network integrity in pathological contexts, potentially alleviating neurodegenerative processes in PD [135,136].

### 3.3. Current Limitations on MSCs Secretome

The use of secretome as a cell-free alternative therapy is advantageous from a clinical translation point of view, since cell-based approaches could still have more ethical issues resulting from the probability (even if low) of tumorigenicity, immune incompatibility, and the possibility of unpredictable pathogen propagation carried by living cells [137,138]. Despite the promising advantages of MSC-derived secretomes, their clinical translation still faces several challenges. These include: (i) establishing standardized methodologies for secretome production to ensure consistency and reproducibility; (ii) defining comprehensive procedures for characterizing bioactive components and elucidating their mechanisms of action; (iii) determining pharmacokinetics, safety, and efficacy in a dose- and disease-specific manner; and (iv) optimizing delivery strategies tailored to therapeutic targets [139].

Further advancements are necessary to enhance its therapeutic efficacy and improve clinical outcomes. In complex disorders such as PD, maximizing the therapeutic potential of the secretome requires targeting specific impaired pathways. With this goal in mind, modulating the secretome composition emerges as a promising strategy to selectively address dysregulated pathways or shift their profile toward a more regenerative state.

### 3.4. Enhancing Mesenchymal Stem Cells Secretome Efficiency

Although MSCs treatments have been gaining relevance in the field of regenerative medicine, their application in clinical settings remains a complex subject. As mentioned above, the high levels of heterogeneity derived from the MSCs’s origin, as well as possible secondary effects that may arise from the broad action of the secretome, are concerns that hinder the translation of these therapies [107,140,141]. Various strategies have been implemented to regulate these variable characteristics, relying on the ability of MSCs to change their phenotype and function according to the surrounding environment. This plasticity enables modifications toward more favorable and desired profiles, which can be designed for specific pathologies, enhancing the therapeutic response and, consequently, the relevance to clinical settings [141].

MSCs priming approaches started to gain relevance after 2003 and reached peak productivity in 2019 [142]. Additionally, a recent publication by the International Society for Cell and Gene Therapy (ISCT) highlighted priming strategies as a promising approach to enhance the basal fitness of MSCs by inducing beneficial phenotypic alterations [143]. The term “cell priming” can be defined as premeditated changes in the microenvironment to achieve a specific function or differentiation through cell activation, molecular signaling, and/or genetic or epigenetic pathways. Initially associated with immunology, priming strategies have been applied to stem cell research, leading to numerous proposals in recent years to enhance MSCs’ function [144]. These approaches can be simplistically divided into two main domains, biophysical and molecular priming, both of which comprise a broad selection of factors. Biophysical priming mainly relies on physical and mechanical stimuli, such as hypoxic conditions [145], three-dimensional and dynamic culturing [146] and electrical stimulation [147]. Conversely, molecular priming presents a more simplistic and direct technique that only consists of the addition of molecules from various origins to the media, impacting cellular biomechanisms. These molecules range from cytokines, growth factors, and hormones to pharmacological and natural compounds that can target specific pathways or have a broader network system [144,148]. Most of these strategies focus on reversing cellular aging and enhancing stemness, as it is the main challenge in the advancement of MSC-based treatments. CRMs have slowly been emerging as promising priming agents, as they mimic the beneficial effects of CR by modifying aging-associated pathways, improving cellular resilience, and reducing senescence [149,150].

## 4. Caloric Restriction Mimetics

CR is one of the most extensively studied and effective interventions for delaying mammalian aging. It is defined as a sustained reduction in caloric intake relative to the amount required for weight maintenance without inducing malnutrition. Importantly, CR protocols ensure that the diet remains nutritionally adequate—providing sufficient energy for metabolic homeostasis and maintaining a high quality in terms of micronutrient and fiber content [151]. It has been proven that not only does it extend life expectancy, but it also delays age-related diseases and decreases their symptoms, and is currently considered one of the best strategies for longevity [152,153]. Many studies in different laboratories have shown that a reduction of 30–60% in calorie intake can increase the lifespan of a wide variety of species [16]. CR is considered a biological stressor since it regulates energy and nutrient-sensing pathways, along with stress-resistance signaling. This involves key regulators, such as AMPK, mTOR, nuclear factor erythroid-related factor 2 (Nrf2), SIRT-1, FOXO, and peroxisome proliferator-activated receptor gamma coactivator-1 alpha (PGC-1α), which are involved in pro-longevity processes, such as autophagy, mitochondrial biogenesis, DNA repair, and the expression of antioxidant and detoxifying enzymes. Although mild to moderate biological stress can produce health benefits, higher stress intensities can be detrimental; thus, such anti-aging strategies must be closely monitored to achieve health benefits [154]. However, adhering to such strict regimens proves to be a challenge in itself, triggering a search for CRM molecules that reach the same pro-longevity benefits of CR without having to alter caloric intake [16].

**Figure 2:**
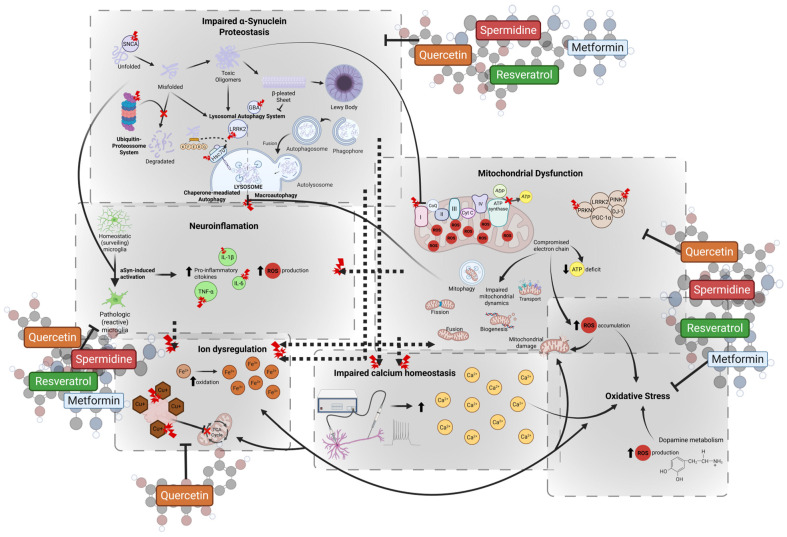
Integrated pathomechanisms of Parkinson’s disease (PD) and points of intervention by caloric restriction mimetics (CRMs). This figure illustrates the key molecular pathways in PD pathogenesis, including asyn aggregation, mitochondrial dysfunction, neuroinflammation, oxidative stress, and ion (metal and calcium) dysregulation. The modulatory effects of four CRMs: quercetin, spermidine, resveratrol, and metformin, which act on multiple, interconnected targets, are overlaid. All four compounds enhance asyn clearance by promoting autophagy and mitophagy via pathways such as PINK1/PRKN and LC3-independent mechanisms. They also attenuate neuroinflammation: quercetin, spermidine, and metformin inhibit NF-κB signaling and shift microglial activation toward an anti-inflammatory state. Mitochondrial quality control is improved through the activation of AMPK, SIRT1, and PGC-1α, which restores energy metabolism and reduces ROS. Oxidative stress is further mitigated by upregulating antioxidant enzymes and, in the case of quercetin, by iron chelation and ferroptosis inhibition. Although calcium is not directly targeted, enhanced mitochondrial and autophagic functions help restore calcium homeostasis. Together, these CRMs converge on PD pathways, proteostasis, inflammation, oxidative stress, and mitochondrial integrity, highlighting their multi-target therapeutic potential. aSyn-αsynuclein; PINK1—phosphatase and tensin homolog induced novel kinase 1; PRKN—Parkin; NF-κB—nuclear factor κ B; AMPK-AMP-activated protein kinase; SIRT1—Sirtuin 1; PGC-1α—Peroxisome proliferator-activated receptor-γ coactivator-1α; ROS—reactive oxygen species. Created in Bio Render (https://www.biorender.com/, accessed on 8 April 2025).

**Figure 3:**
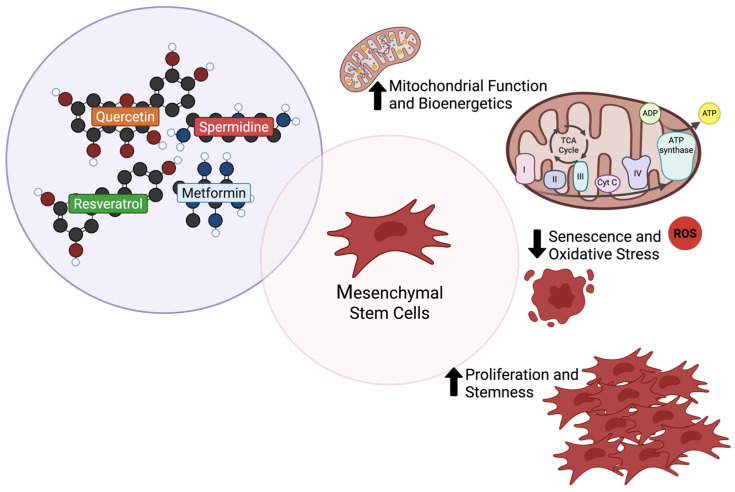
Caloric restriction mimetics (CRMs) priming effects on mesenchymal stem cells (MSCs). Collectively, CRMs—quercetin, spermidine, resveratrol, and metformin—enhance mitochondrial bioenergetics, mitigate oxidative damage, suppress cellular senescence, and promote both proliferative capacity and stemness. These benefits are mediated by the activation of key molecular pathways, including antioxidant defense mechanisms and autophagy regulation. Preservation of mitochondrial integrity and promotion of proteostasis contribute to improved cell viability, delayed replicative aging, and enhanced differentiation potential, particularly toward neuroectodermal lineages. These findings underscore the potential of CRMs as priming agents to optimize MSC fitness and therapeutic efficacy in regenerative medicine. ROS—reactive oxygen species. Created in Bio Render (https://www.biorender.com/, accessed on 8 April 2025).

The definition of CRMs may vary in the literature, as many definitions seem to arise due to the fast-evolving nature of the field and the broad effects attributed to CR. However, the most widely accepted concept characterizes CRMs as bioactive molecules that mimic the key benefits of CR, including lifespan extension and reduction in age-associated diseases. These compounds enhance autophagy, reduce oxidative stress and damage, promote mitochondrial adaptation, increase stress response, and maintain cellular cycle, mainly by targeting the insulin, TOR, AMPK, and SIRT pathways [16,17,18]. Importantly, emerging evidence shows that CRMs can also exert modulatory effects on MSCs, particularly by reshaping their secretome profile. This includes the upregulation of neurotrophic and anti-inflammatory factors and the downregulation of pro-inflammatory mediators, which together foster a more regenerative and neuroprotective environment [155,156,157]. Such changes are especially relevant for PD, where MSC-derived secretomes enhanced by CRMs may support dopaminergic neuron survival, reduce neuroinflammation, and restore mitochondrial function. Going forward, we will dive deeper into key CRMs, quercetin, spermidine, resveratrol, and metformin, unraveling their intricate interactions with PD (Figure 2 and Table 1) and MSCs (Figure 3 and Table 2). Our focus is on their potential as powerful therapeutic enhancers, paving the way for innovative treatment strategies.

### 4.1. Quercetin

Quercetin is one of the most abundant dietary flavonoids, accounting for 60–75% of flavonoid intake. It is most abundantly found conjugated to sugars as glycosylated forms, with the aglycone conformation being less abundant in nature. Quercetin is nutritionally available in various sources, such as onions, shallots, broccoli, asparagus, green peppers, tomatoes, berries, green tea, and wine [185]. This low-toxicity compound exhibits antioxidant [186], anti-inflammatory [187] and anti-senescent [188] properties; however, its poor and inconsistent bioavailability, solubility, permeability, and instability have hindered its usage [189]. Despite this, recent reports indicate that quercetin exerts protective effects against age-related diseases. At the molecular level, quercetin has been shown to exert multi-target effects. It is hypothesized that these effects occur mainly through SIRT1 regulation, influencing key pathways such as PI3K/Akt, NF-κB, and Nrf2/ heme oxygenate 1 (HO-1) [189,190,191].

#### 4.1.1. Quercetin and Parkinson’s Disease

Several studies have demonstrated that quercetin is able to modulate critical pathways in PD, acting as a neuroprotective agent. In a 1-methyl-4-phenyl-1,2,3,6-tetrahydropyridine (MPTP)-induced in vitro model of PD, quercetin exhibited neuroprotective effects by decreasing ferroptosis through Nrf2-dependent pathways. By upregulating Nrf2, quercetin modulated the levels of several key players in ferroptosis induction, such as GPX4, malonaldehyde (MDA), iron content, nuclear receptor coactivator 4 (NCOA4), and solute carrier family 7 member 11 (SLC7A11) [158]. In 6-OHDA-treated cells and rats, it enhanced *PINK1*/*Parkin* expression, preventing neuronal loss and behavioral deficits [159]. Additionally, in a *Caenorhabditis elegans* model, quercetin induced mitophagy, leading to a reduction in oxidative stress, mitochondrial damage, and asyn expression/accumulation [159].

Quercetin also activated PGC-1α, boosting mitochondrial biogenesis and bioenergetic capacity in dopaminergic neurons. Furthermore, it influenced cell survival pathways by activating protein kinase D1 (PKD1) and Akt, promoting neuronal survival and resistance to 6-OHDA. It also increased cAMP response-element binding protein (CREB) phosphorylation and upregulated BDNF expression, supporting neuronal growth and plasticity [161].

Beyond its neuroprotective actions, quercetin exhibits potent anti-inflammatory effects in PD models by inhibiting NF-κB activation, leading to reduced levels of pro-inflammatory cytokines, such as TNF-α and IL-6, thereby limiting neurotoxic inflammation [162]. Additionally, quercetin modulated apoptosis by downregulating pro-apoptotic proteins like B-cell lymphoma 2-associated X (Bax) and caspase-3, and upregulating the anti-apoptotic protein B-cell lymphoma 2 (Bcl-2), thereby protecting neurons from toxin-induced death [160].

#### 4.1.2. Quercetin and Mesenchymal Stem Cells

As a priming agent, quercetin appears to support the viability and functionality of MSCs. In MSCs derived from human exfoliated deciduous teeth (SHEDs), quercetin affected cellular viability, mitochondrial function, fatty acid composition, and the expression of oxidative stress and SIRT genes in a passage- and dosage-dependent manner. In younger SHEDs, it enhanced metabolic activity and mitochondrial respiration, while enhancing the levels of lauric and myristic acids and reducing oleic acid levels, potentially impacting cellular membrane properties and overall function. In older SHEDs, quercetin preserved mitochondrial function and enhanced stearic acid levels, a lipid that enhances endogenous antioxidant enzymes, suggesting the activation of oxidative stress defense mechanisms, often associated with senescence. In these later passages, the modulation of oxidative stress gene expression and SIRT levels supports the hypothesis that quercetin may trigger molecular cascades that counteract age-related declines in MSCs’ viability and function [176].

Quercetin has also been shown to modulate the inflammatory profile of MSCs by downregulating p-Akt/p-IκB expression, upregulating toll-like receptor 3 (TLR-3), and inducing higher anti-inflammatory factor levels in Human Umbilical Cord MSCs (hUC-MSCs) [177]. Therefore, the reduction in the phosphorylated levels of Akt and IκB, which reflects reduced activity, decreases the consequent inflammatory cascades. Additionally, the upregulation of TLR-3 further amplifies the secretion of NO, IDO, and IL-6. While IL-6 is often linked to pro-inflammatory mechanisms, it has a dual functionality [192]. Given its co-secretion with potent antioxidant molecules, this suggests that, in this context, IL-6 may contribute to an immunosuppressive and protective role [177].

### 4.2. Spermidine

Spermidine is a natural polyamine present in all organisms, including humans, as well as in various vegetables, fruits, and meats, and is ingested through the diet. As a polyamide, it plays a crucial role in cellular growth, proliferation, and tissue regeneration by stabilizing DNA and RNA, thereby supporting essential cellular functions and repair mechanisms [193,194]. Spermidine also displays antioxidant [195] and anti-inflammatory [196] properties, as well as the ability to modulate mitochondrial function, proteostasis, and chaperone activity [197]. The pleiotropic benefits of spermidine are primarily attributed to its ability to induce autophagy, which is considered its most important mechanism of action [193,197].

Spermidine has also been shown to extend both life and healthspan, making it a promising candidate for clinical trials due to its high efficacy and low toxicity. As a naturally occurring polyamine, its levels decline with age; however, supplementation has been linked to reversing age-related memory impairment and protecting neurons from autoimmune-driven demyelination, further highlighting its potential [193].

#### 4.2.1. Spermidine and Parkinson’s Disease

As an endogenous molecule, spermidine has been linked to PD. A study exploring the polyamine profile in patients with PD revealed a dysfunction in the conversion of spermidine to spermine, resulting in a reduced spermine/spermidine ratio in an age-independent manner. Interestingly, while this ratio typically declines gradually with age in healthy individuals, in patients with PD, the reduction appears to be independent of age. Additionally, N1,N8-diacetylspermidine, a byproduct of spermidine acetylation correlated with disease severity, has been proposed as a medication-independent biomarker of PD [198].

Spermidine has also been extensively studied as a potential therapeutic agent for PD in several model organisms. In *Drosophila melanogaster* PD models, spermidine supplementation restored the lifespan of these models compared to that of wild-type flies and mitigated motor dysfunction. This neuroprotective effect was accompanied by an increase in Atg8a-II levels (LC3 homolog), suggesting that autophagy activation is a key protective mechanism [163]. Similarly, in *C. elegans* PD models, spermidine administration was able to reduce asyn expression and aggregation, improve motor ability, and chemical tropism-mediated learning ability [164], and rescue asyn-induced neuronal degeneration [163]. Mechanistically, spermidine modulated autophagy-related pathways by upregulating *bec-1* (*BCEN1* homolog) and downregulating *sqst-1* (sequestosome (*SQSTM*) homolog) mRNA levels. These effects were abolished in *PINK1* and *PDR-1* (*PRKN* homologous) knockout nematodes, indicating the dependence of spermidine neuroprotection on mitophagy pathways [164].

In mammalian PD models, spermidine exhibited similar protective effects. In a rotenone-induced PD rat model, spermidine treatment effectively counteracted rotenone’s effects by restoring motor function, alleviating oxidative stress, decreasing pro-inflammatory cytokine levels (TNF-α, IL-1β, IL-6), and replenishing striatal catecholamines and gamma-aminobutyric acid (GABA) concentrations [166]. Likewise, in an MPTP-induced PD mice model, spermidine pre-administration modulated microglial function by reducing M1 microglial (pro-inflammatory phenotype) markers and enhancing M2 microglial (anti-inflammatory phenotype) markers. This effect was associated with the inhibition of NF-κB, P65, signal transducer and activator of transcription 1 (STAT1), and P38 mitogen-activated protein kinase (MAPK) activation, while promoting the phosphorylation of STAT6 and reducing IL-1β, IL-6, and TNF-α expression. These findings suggest that spermidine facilitates a neuroprotective shift in microglial polarization, further reinforcing its potential as a PD therapeutic [165].

#### 4.2.2. Spermidine and Mesenchymal Stem Cells

The role of spermidine as a priming agent in MSCs remains largely unexplored, with most studies focusing on its endogenous levels rather than its supplementation effects. It was shown that the spermidine and spermine levels significantly decline in MSCs undergoing osteogenesis. However, elevated endogenous polyamine levels induce cytoplasmic vacuolization, disrupt mitochondrial function, and suppress matrix mineralization during osteoblastogenesis. These effects were reversed by difluoromethylornithine (DFMO), an inhibitor of ornithine decarboxylase (ODC1), which is an upstream enzyme in the polyamine synthesis pathway. This suggests that maintaining balanced spermidine levels is critical for osteogenic differentiation and MSCs homeostasis [199].

Nonetheless, a study using hUC-MSCs demonstrated that spermidine supplementation increased proliferation rates, reduced senescence-associated β-galactosidase (SA-β-gal) activity, and downregulated senescence markers, including phosphorylated P53 (p-P53-ser15), total P53, and P21, particularly in late-passage cells. In addition, Ki67, a key molecule associated with proliferation efficiency, was also significantly enhanced, along with SIRT3, a mitochondrial deacetylase known for its role in reducing ROS and promoting mitochondrial function. Spermidine also facilitated the maintenance of adipogenic and osteogenic differentiation, suggesting its role in delaying replicative senescence. The depletion of these protective effects in SIRT3 knockout MSCs further confirms that spermidine anti-senescent mechanisms are dependent on SIRT3 modulation [178]. Together, these findings highlight the dual role of spermidine in MSC biology: while excessive endogenous levels can impair osteogenesis, controlled exogenous supplementation may enhance MSC proliferation, mitigate senescence, and preserve differentiation potential. Further research is warranted to optimize spermidine-based strategies for improving MSC-based regenerative therapies.

### 4.3. Resveratrol

Resveratrol is a natural polyphenol that was first isolated in 1939 by Takaoka from the plant Veratrum grandiflorum [200]. It is highly concentrated in the skin of red grapes and, consequently, is present in wine. However, it can also be found in over 70 plant species, including tea, berry fruits, pomegranates, nuts, and dark chocolate [201]. It is a secondary metabolite that plays a role in the mechanisms of protection against environmental stressors and pathogenic attacks in plants [202]. Many studies have highlighted resveratrol’s pleiotropic effects, such as amelioration of oxidative stress, suppression of inflammation, regulation of mitochondrial function, inhibition of apoptosis, and reduction of DNA damage [203]. These protective functions have been attributed to the activation of SIRT1, a key regulator of cellular longevity and stress resistance. However, the direct activation of SIRT1 by resveratrol has been debated, with alternative hypotheses suggesting a mechanism mediated through AMPK activation, which in turn influences SIRT1 activity [204].

Despite the fact that resveratrol molecular mechanisms still remain elusive, it has been consistently associated with increased lifespan in various organisms, including *Saccharomyces cerevisiae* [205], *C. elegans* [206], *D. melanogaster* [207], *Nothobranchius furzeri* (a short-lived fish species) [208], and mice in high-calorie diet contexts [209]. Its ability to extend lifespan is thought to be linked to its role in promoting cellular resilience, modulating energy metabolism, and enhancing stress resistance in cells.

However, despite promising findings from in vitro and in vivo studies, translating resveratrol’s benefits to humans has yielded inconsistent results. One major limitation is its poor bioavailability, as resveratrol undergoes rapid metabolism and clearance, potentially diminishing its effectiveness in clinical settings [210]. Strategies to enhance its bioavailability, such as the use of nanoparticle formulations, structural analogs, and combination therapies with bioenhancers like piperine, are currently being explored to improve its therapeutic potential. Further research is needed to fully elucidate the mechanisms of action of this compound and to optimize its clinical applications.

#### 4.3.1. Resveratrol and Parkinson’s Disease

Notwithstanding the translational challenges in clinical settings, resveratrol remains a promising therapeutic candidate for PD, with multiple studies demonstrating its neuroprotective effects across various models. In a study using fibroblasts derived from two patients with early-onset PD with distinct *PARK2* mutations, resveratrol treatment activated the AMPK and SIRT1 pathways, resulting in increased mRNA expression of PGC-1α target genes. This upregulation was associated with mitochondrial oxidative function, as evidenced by increased complex I and citrate synthase activities, elevated basal oxygen consumption, and higher mitochondrial ATP production. In contrast, resveratrol reduced lactate content, suggesting a metabolic shift from glycolytic to oxidative metabolism. Additionally, it promotes autophagic flux through the activation of an LC3-independent pathway, further supporting its role in mitochondrial quality control [167].

Similarly, in an in vitro PD model using rotenone-treated SH-SY5Y cells, resveratrol pretreatment decreased rotenone-induced apoptosis by enhancing SIRT1 expression and AMPK phosphorylation. This resulted in the depletion of P53 and acetylated histone H3 lysine 9 (H3K9) expression, suggesting that resveratrol is able to counteract cell proliferation arrest and support cellular survival mechanisms [168]. In another study performed in a *D. melanogaster* MPTP-induced PD model, resveratrol reduced cell death, histological alterations, and behavioral deficits. It restored catalase, glutathione-S-transferase, and acetylcholinesterase activities while modulating NO and H_2_O_2_ levels, highlighting its antioxidative and neuroprotective effects [169]. In a 6-OHDA-induced PD rat model, improved motor function and increased body weight were observed in the resveratrol-treated group, as well as an increased number of TH-positive cells in the SNpc. In the midbrain, resveratrol decreased Bax and active caspase-3 levels, while enhancing Bcl-2, PI3K-110α, and p-Akt Ser473 expression, effectively delaying apoptosis. These findings suggest that resveratrol exerts its protective effects by modulating apoptotic signaling and enhancing neuronal survival [170].

Collectively, these studies underscore resveratrol’s multifaceted neuroprotective mechanisms in PD, including mitochondrial enhancement, metabolic reprogramming, autophagy modulation, antioxidative defense, and anti-apoptotic signaling. However, further in vivo and clinical research is necessary to optimize its therapeutic potential and overcome limitations, such as low bioavailability.

#### 4.3.2. Resveratrol and Mesenchymal Stem Cells

As mentioned earlier, resveratrol’s clinical application remains unreliable, limiting its viability as a standalone therapy. However, its biological properties and effectiveness in vitro have positioned it as a promising enhancer of cell-based therapy. Its impact on AMPK, SIRT, autophagy, and oxidative stress pathways makes it an interesting candidate for improving MSCs’ stemness, self-renewal, and differentiation potential [148,211]. A study demonstrated that resveratrol was able to reduce phosphorylated extracellular signal-regulated kinase (ERK) in early passage MSCs, supporting their maintenance and viability. However, in late-passage and SIRT1-knockdown MSCs, resveratrol had the opposite effect, increasing ERK activation and consequently stimulating β-catenin activity, promoting ROS production, and inducing senescence. This suggests that resveratrol’s ability to enhance MSCs’ stemness and viability is correlated with the endogenous levels of SIRT1 [179]. Another study using hUC-MSCs observed that prolonged exposure to low doses of resveratrol increased SIRT1 levels and reduced P53 and P16 expression, facilitating self-renewal by enhancing viability and proliferation, while higher doses exerted the opposite effect. Neuronal-lineage differentiation was also facilitated by enhanced levels of the neuronal markers βIII-tubulin and neuron-specific enolase (NSE) and pro-neuronal transcription factors neurogenin 2 (Ngn2) and Mash1, as well as reduced levels of nestin and Ngn1, resulting in resveratrol-induced morphological changes [180]. Resveratrol-induced neuronal cell differentiation has also been observed in dental pulp-derived MSCs (DPSCs), where it increased the expression of neuron-specific marker genes such as nestin, musashi, and neurofilament M (NF-M) [181]. Additionally, resveratrol’s ability to modulate oxidative stress and autophagic flux may further support MSCs’ survival and differentiation in neurodegenerative disease models, making it a promising adjunct in regenerative medicine. While these findings highlight resveratrol’s potential in MSC-based therapies, further studies are needed to refine its application, optimize dosage strategies, and determine its long-term effects on MSCs’ fate and function.

### 4.4. Metformin

Metformin is a synthetic biguanide derived from galegine, a natural product present in *Galega officinalis*, used in herbal medicine in medieval Europe. Unlike most modern pharmacological compounds, metformin’s structure was not designed to target specific pathways, as it is derived from a natural product and has broad molecular interactions [212]. Despite being prescribed for over 60 years and used every day by over 150 million people as the first-line anti-hyperglycemic treatment for type 2 diabetes, its precise mechanisms of action remain incompletely understood.

Metformin primarily acts as an insulin sensitizer, reducing insulin demand and consequently modulating IGF-1 levels. This IGF-1 modulation is linked to metformin’s activation of AMPK and inhibition of mTOR signaling pathways, sharing the CR molecular pathways [213]. Beyond its anti-diabetic action, metformin has been proven to be effective in targeting aging-related pathologies [214] and extending the lifespan of *C. elegans* [215] and mice [216].

#### 4.4.1. Metformin and Parkinson’s Disease

As mentioned above, metformin has gained recognition for its ability to attenuate the various molecular hallmarks of aging. Recently, its potential as a therapeutic agent in treating age-related comorbidities beyond diabetes mellitus, including PD, has gained increasing attention [217]. Studies on metformin’s interaction with PD have emerged, reporting that it plays a significant role in PD pathology [218]. In an in vitro PD model using SH-SY5Y cells, metformin pretreatment improved the viability of rotenone-treated cells by inhibiting caspase-3 activation and reducing intracellular and mitochondrial ROS levels. Additionally, metformin upregulates glutathione (GSH), cytosolic and mitochondrial superoxide dismutase (SOD), PGC-1α, and Nfr2, providing antioxidant effects and enhancing mitochondrial function [171]. Similarly, in a *C. elegans* PD model, metformin was able to effectively reduce 6-OHDA-induced neurodegeneration and restore food-sensing behavior without impacting the development of the nematodes. It also inhibited asyn aggregation and upregulated catalase-2 (*cat-2*) and *sod-3* genes, which are related to DA synthesis and free-radical scavenging, respectively [173]. Several studies in PD models have provided evidence that metformin treatment can prevent mitochondrial dysfunction and neurodegeneration by improving mitochondrial membrane potential and increasing ATP production, further supporting its role in enhancing mitochondrial quality control mechanisms [172,174,219,220].

A study using an MPTP-induced PD mouse model demonstrated that metformin improved motor function, increased the number of TH-positive neurons, and elevated striatal DA levels. Additionally, it reduced microglial activation and asyn accumulation, accompanied by increased levels of methylated protein phosphatase 2A (PP2A), a phosphatase related to asyn dephosphorylation, suggesting metformin-induced mechanisms against asyn toxicity. Molecular analysis revealed that metformin induced the activation of AMPK, Akt, and ERK downstream pathways, inhibition of mTOR signaling, and upregulation of BDNF, all of which contribute to its neuroprotective effects [175]. Together, these findings suggest that metformin mitigates PD pathology by promoting mitochondrial function, enhancing autophagy, reducing oxidative stress, and modulating neuroinflammatory responses. However, further clinical studies are required to determine its efficacy and safety in patients with PD.

#### 4.4.2. Metformin and Mesenchymal Stem Cells

Metformin has also been gaining attention in the field of regenerative medicine, as recent studies suggest its potential to enhance MSCs’ potency through activation of osteogenic and neuronal differentiation as well as increased levels of stemness markers [148]. This compound demonstrated senomorphic properties by reducing replicative senescence and apoptosis in MSCs, maintaining decreased levels of β-gal, and the presence of DNA-synthesizing cells through prolonged in vitro cultivation. Proteomic analysis of MSCs secretome revealed that metformin-treated MSCs secrete molecules involved in α-adrenergic signaling (which regulates physiological secretory activity), detoxification pathway, and aspartate degradation (which optimizes energy production). Additionally, metformin-treated MSCs exhibited upregulated levels of key antioxidant proteins, including SOD1, SOD2, CAT, glutaredoxin (GLRX), and glutathione S-transferase (GST), suggesting that metformin supplementation reduced the impairment of MSCs’ functions through ROS scavenging mechanisms [182]. Another proteomic analysis of MSCs’ secretome revealed that metformin enhanced EVs production and secretion through autophagy-related pathways. Moreover, EVs derived from metformin-primed MSCs had more functional relevance than those from the control groups, indicating that metformin also improves the quality of the secretome content [183]. Additionally, metformin has been shown to support MSCs differentiation into neuronal lineages. It promotes neurogenic commitment by increasing the expression of neuron-specific genes, such as βIII-tubulin and MAP2, while modulating key signaling pathways involved in neural differentiation [184].

These findings underscore metformin’s potential to enhance MSC-based regenerative therapies by improving cell survival, reducing senescence, boosting differentiation potential, and enriching the MSC-secretome. Future research should focus on optimizing dosing strategies and evaluating the long-term effects to harness the full potential of metformin stem cell applications.

### 4.5. Strategic Priming of Mesenchymal Stem Cells with Caloric Restriction Mimetics

Despite these promising insights, several challenges remain in optimizing MSCs priming with CRMs. Key questions include the duration and stability of CRM-induced functional enhancements, effects of repeated or combined stimuli, and impact of donor variability and cell source. Furthermore, clinical translation is hindered by concerns such as immunogenicity, tumorigenicity, and the lack of standardized, GMP-compliant protocols.

Priming MSCs with CRMs can be performed by exposing the cells to low, non-toxic concentrations of compounds such as resveratrol, quercetin, spermidine, or metformin for 24 to 72 h under standard or hypoxic conditions, ensuring solubility, cell viability, and efficient uptake [221]. However, further research is needed to clarify the in vivo effects, evaluate the viability of cryopreserved primed MSCs, and assess the long-term safety and efficacy of these strategies. Standardization of potency assays and optimization of CRMs’ usage, particularly regarding concentration, timing, and delivery, are crucial for improving the reproducibility of MSC-based therapies. Understanding the pharmacokinetics and pharmacodynamics of CRMs in the context of MSC biology is essential. For example, dose–response and time–response studies are key to maximizing efficacy while avoiding cytotoxicity or unwanted differentiation, especially as compounds like quercetin, spermidine, and metformin can exhibit biphasic effects depending on their concentrations [177,178,201,222,223].

Solubility and bioavailability remain significant challenges, particularly for hydrophobic polyphenols, such as resveratrol. Nanoparticle-based delivery systems and liposomal formulations are also needed to improve CRM stability and targeted intracellular delivery.

Combinatorial priming strategies, in which CRMs are paired with biomaterials, hypoxic preconditioning, or MSC-derived secretomes, may produce synergistic effects. These approaches better replicate the complex in vivo environment and could enhance the therapeutic efficacy of MSC upon transplantation. Donor heterogeneity and tissue-specific differences in MSCs’ responsiveness must also be carefully considered. Moreover, the long-term tumorigenic potential of primed MSCs requires a rigorous evaluation. Although CRMs like quercetin and resveratrol exhibit anti-cancer properties, their effects on genomic stability, telomerase activity, and epigenetic modifications remain insufficiently understood [210,224]. Longitudinal in vivo studies and next-generation sequencing approaches are essential for identifying potential risks before clinical application.

In summary, CRMs offer a promising avenue for enhancing MSC stress resilience, immunomodulatory function, and regenerative potential. However, realizing their full clinical potential will require carefully designed, source-specific priming protocols, robust standardization, and comprehensive safety validation. If optimized, CRM-based priming could enable a new generation of MSC therapies with improved outcomes for treating complex inflammatory and degenerative diseases.

## 5. Conclusions and Future Perspectives

CRMs, including quercetin, spermidine, resveratrol, and metformin, have emerged as promising agents capable of modulating aging-related pathways. While these compounds show potential in mitigating neurodegeneration, their therapeutic impact remains limited due to challenges in bioavailability, delivery, and the inability to fully restore impaired cellular functions.

In addition to their direct neuroprotective effects, CRMs enhance the regenerative potential of MSCs by improving their viability, paracrine activity, and immunomodulatory properties, making them attractive candidates for advancing cell-based therapies. However, research on CRM-driven MSCs priming is still in its infancy, and whether CRMs can specifically target PD-related pathways in MSCs remains an open question. Most existing studies have focused on their ability to delay senescence and influence differentiation, rather than their direct impact on PD pathology.

A deeper understanding of how CRMs modulate MSCs could unlock a novel synergistic strategy to enhance the therapeutic potential of MSC-derived secretome for PD treatment. Optimized preconditioning protocols may enable the development of an enhanced secretory profile [221], leading to improved neuroprotection, reduced neuroinflammation, restored proteostasis and mitochondrial function, and enhanced oxidative stress clearance. While current evidence supports the benefits of CRMs and MSCs individually, the potential of their combined application remains largely unexplored. Future research should focus on unraveling the molecular mechanisms underlying CRM-induced MSCs priming, optimizing dosage and administration strategies, and ensuring long-term safety and efficacy in both preclinical and clinical settings. In this context, several clinical limitations must be addressed before effective therapies can be developed. Challenges include determining the optimal dosing regimens for both CRMs and MSC-based treatments, as excessive or prolonged exposure may lead to cytotoxicity or undesired immunological effects. Additionally, issues related to bioavailability, pharmacokinetics, and targeted delivery of CRMs limit their therapeutic efficiency and must be overcome through advanced formulation technologies. Standardization of MSCs’ preconditioning protocols is also urgently needed to ensure reproducibility, consistency, and regulatory compliance across clinical applications. Addressing these limitations is critical for developing safe, effective, and scalable therapeutic strategies that harness the full regenerative potential of CRMs and MSCs in PD. In conclusion, CRM-based MSCs priming represents a groundbreaking and highly targeted therapeutic approach for PD. By bridging the gap between metabolic interventions and regenerative medicine, this strategy could lead to transformative advancements in PD management, offering new hope for the treatment of neurodegenerative disease.

## Figures and Tables

**Table 1:** Main molecular mechanisms and neuroprotective effects of caloric restriction mimetics (CRMs) in experimental models of Parkinson’s disease (PD).

CRM	Type of Study	Model System	PD Model	Effects
Quercetin	In vitro	SH-SY5Y	MPTP	Reduced apoptosis, MDA, NCOA4; Upregulated GPX4, Nrf2 and SLC7A11 [158].
		PC12	6-OHDA	Enhanced *PINK1/Parkin* expression; Prevented neuronal loss [159].
			H_2_O_2_	Downregulated Bax and caspase-3; Upregulated Bcl-2; Reduced apoptosis [160].
		MN9D	-	Activated PGC-1α, PKD1, Akt, and CREB; Upregulated BDNF; Increased basal OCR and ATP-linked respiration [161].
			6-OHDA	Toxin resistance [161].
	In vivo	*C. elegans*	Transgenic neuronal mt-Rosella	Induced mitophagy; Reduced oxidative stress, mitochondrial damage, and asyn accumulation [159].
		Rat	6-OHDA	Enhanced *PINK1/Parkin* expression; Decreased neuronal loss and behavioral deficits [159].
			Rotenone	Reduced TNF-α, IL-1β, and IL-6; Attenuated motor deficits; Improved biochemical and neurotransmitter alterations [162].
Spermidine	In vivo	*C. elegans*	Transgenic asyn expression	Decreased neuronal degeneration. (UA44 strain) [163].
				Increased mean lifespan, locomotor capacity, and chemotaxis-based cognitive ability; Reduced asyn; Upregulated *bec-1*; Downregulated *sqst-1*. (NL5901 strain) [164].
		*D. melanogaster*	Transgenic asyn expression exposed to manganese	Increased mean lifespan and Atg8a-II levels; Decreased motor deficits [163].
		Mouse	MPTP	Reduced IL-1β, IL-6, TNF-α, and M1 microglial markers (CD16, CD32, CD86); Increased M2 microglial markers (Arg-1, CD206, Ym1), STAT6 activation, behavioral scores, TH-positive neurons, and TH expression in SN; Decreased activation of NF-κB p65, STAT1, and p38 MAPK [165].
		Rat	Rotenone	Decreased weight loss, motor dysfunction, and MDA, nitrite, TNF-α, IL-1β, IL-6, and glutamate levels; Increased GSH, GABA, and norepinephrine, dopamine, serotonin, and respective metabolites [166].
Resveratrol	In vitro	Fibroblasts (early-onset patients)	*PARK2* heterozygous mutations	Increased OCR, ATP production, complex I and citrate synthase activity, relative mitochondrial DNA content, AMPK activation, NAD+/NADH ratio, PGC-1α, mitochondrial transcriptional factor A, cytochrome c, cyclooxygenase 1, SOD2, CAT, SIRT1, and LC3-independent macroautophagy; Decreased mitochondrial ROS and acetylated-H3 [167].
		SH-SY5Y	Rotenone	Decreased cell death, Bax, apoptotic cells, P53, cells in G0/G1 phase and acetylated H3K9; Increased Bcl-2, AMPK activation, SIRT1, cells in G2/M phase and tri-methylated H3K9 [168].
	In vivo	*D. melanogaster*	MPTP	Increased climbing rate, acetylcholinesterase, CAT and GSH activity, emergence of flies, and cell viability; Reduced H_2_O_2_ and NO [169].
		Rat	6-OHDA	Improved motor function and body weight; Increased Bcl-2, PI3K-110α, p-Akt Ser473, and TH-positive cells in SN; Decreased Bax and active caspase-3; Delayed apoptosis [170].
Metformin	In vitro	SH-SY5Y	Rotenone	Improved cell viability; Inhibited caspase-3 activation; Reduced intracellular and mitochondrial ROS; Increased GSH activity, cytosolic and mitochondrial SOD, PGC-1α, and Nrf2 levels [171].
		N27	MPTP	Increased mitochondrial bioenergetics capacity, TFAM, and mitochondrial DNA content. Reduced mitochondrial fragmentation and dopaminergic neuronal degeneration [172].
	In vivo	*C. elegans*	6-OHDA	Reduced neurodegeneration and asyn aggregation; Restored food-sensing behavior; Upregulated cat-2 and sod-3 gene expression [173].
			*b-cat1* knockdown	Reduced mitochondrial respiration to control levels. Improved motor function and neuronal viability [174].
		Mouse	MPTP	Improved motor function; Increased TH-positive neurons, striatal dopamine, methylated PP2A levels, and BDNF expression; Reduced microglia activation, asyn accumulation, and mTOR signaling; Activated AMPK, Akt, and ERK [175].

MDA—malonaldehyde; NCOA4—iron content and nuclear receptor coactivator 4; GPX4—glutathione peroxidase 4; Nrf2—nuclear factor erythroid-related factor 2; SLC7A11—solute carrier family 7; PINK1—phosphatase and tensin homolog induced novel kinase 1; Bax—B-cell lymphoma 2 associated X protein; Bcl-2—B-cell lymphoma 2; PGC-1α—peroxisome proliferator-activated receptor-γ coactivator-1α; PKD1—protein kinase D1; Akt—protein kinase B; CREB—cAMP response-element binding protein; BDNF—brain-derived neurotrophic factor; OCR—oxygen consumption rate; TNF-α—tumor necrosis factor α; IL—interleukin; Arg-1—arginase-1; Ym1—Chitinase-like protein 3; STAT—signal transducer and activator of transcription; TH—tyrosine hydroxylase; SN—substantia nigra; NF-κB—nuclear factor κ B; MAPK—mitogen-activated protein kinase; GSH—glutathione; GABA—gamma-aminobutyric acid; AMPK—AMP-activated protein kinase; SOD—superoxide dismutase; CAT—catalase; SIRT—sirtuin; ROS—reactive oxygen species; H3K9—histone H3 lysine 9; NO—nitric oxide; PI3K—phosphoinositide 3-kinase; TFAM—mitochondrial transcription factor A; PP2A—protein phosphatase 2A; mTOR—mechanistic target of rapamycin; ERK—extracellular signal-regulated kinase.

**Table 2:** Mechanistic insights into the actions of caloric restriction mimetics (CRMs) on the function of mesenchymal stem cells (MSCs).

CRM	MSCs Cell Source	Condition	Effects
Quercetin	SHEDs	Early Passages (Passage 5)	Increased metabolic activity, mitochondrial respiration, and levels of lauric and myristic acids; reduced levels of oleic acid [176].
		Later Passages (Passage 16)	Preserved mitochondrial function; increased levels of stearic acid; modulated expression of oxidative stress genes and sirtuins [176].
	hUC-MSCs	- (Passage 3–5)	Reduced activation of Akt and IκB; increased expression of TLR-3; enhanced production of NO, IDO, and IL-6 [177].
Spermidine	hUC-MSCs	Later Passages (Passage 26)	Increased proliferation, Ki67, SIRT3; Reduced SA-β-gal, p-P53, P53, P21 and ROS; Improved mitochondrial function; Maintained adipogenic/osteogenic potential; SIRT3 knockout abolished these benefits—indicating SIRT3-dependency [178].
Resveratrol	hBM-MSCs	Early Passages (Passage 1–3)	Reduced ERK activation [179].
		Late Passages (Passage 9–10)/SIRT1 knockdown	Increased ERK, β-catenin, ROS, and senescence; Indicates SIRT1-dependent dual effect [179].
	hUC-MSCs	- (Passage 4)	Increased SIRT1, βIII-tubulin, NSE, Ngn2 and Mash1; Decreased P53, P16, Nestin and Ngn1; Induced morphological changes [180].
	DPSCs	- (Passage 3–5)	Increased Nestin, Musashi, and NF-M [181].
Metformin	ASCs	- (Passage 3)	Supported long-term viability; Reduced senescence, apoptosis, and β-gal; Increased DNA synthesis, SOD1/2, CAT, GLRX, GST, and secretion of molecules involved in α-adrenergic signaling, detox, and aspartate degradation [182].
	hBM-MSCs	- (Passage 2–3)	Increased EV production via autophagy-related pathways and secretome functional relevance [183].
		- (Passage 7)	Increased βIII-tubulin, MAP2, and key neurogenic signaling [184].

Akt—protein kinase B; TLR-3—toll-like receptor 3; NO—nitric oxide; IDO—indoleamine 2,3-dioxygenase; IL—interleukin; SIRT—sirtuin; SA-β-gal—senescence-associated β-galactosidase; ROS—reactive oxygen species; ERK—extracellular signal-regulated kinase; NSE—neuron-specific enolase; Ngn—neurogenin; Mash1—Achaete-scute homolog 1; NF-M—neurofilament M; SOD—superoxide dismutase; CAT—catalase; GLRX—glutaredoxin; GST—glutathione S-transferase; EV—extracellular vesicle; MAP2—microtubule-associated protein 2.

## Data Availability

Not applicable.

## Abbreviations

The following abbreviations are used in this manuscript:

Akt	protein kinase B
AMPK	adenosine-monophosphate-activated protein kinase
aSyn	α-synuclein
ATG	autophagy related
Bax	B-cell lymphoma 2 associated X protein
BBB	blood−brain barrier
Bcl-2	B-cell lymphoma 2 protein
BDNF	brain-derived neurotrophic factor
BECN1	beclin 1
CAT	catalase
CCL4	chemokine ligand 4
CFL1	cofilin 1
CLU	clusterin
CMA	chaperone-mediated autophagy
CR	caloric restriction
CREB	cAMP response-element binding protein
CRMs	caloric restriction mimetics
CST3	cystatin C
DBS	deep brain stimulation
DFMO	difluoromethylornithine
DMT1	divalent metal transporter 1
DPSCs	dental pulp-derived MSCs
ECM	extracellular matrix
EGF	epidermal growth factor
ERK	extracellular signal-regulated kinase
EVs	extracellular vesicles
FGF-9	fibroblast growth factor 9
FOXO	forkhead box O
GABA	gamma-aminobutyric acid
GABARAPL1	GABA type A receptor-associated protein like 1
GAD	glutamic acid decarboxylase
GBA	glucosylceramidase beta
GDNF	glial-derived neurotrophic factor
GLRX	glutaredoxin
GPX4	glutathione peroxidase 4
GSH	glutathione
GST	glutathione S-transferase
H3K9	histone H3 lysine 9
hBM	human bone marrow-derived
HGF	hepatocyte growth factor
HO-1	heme oxygenate 1
HSC70	heat shock cognate 70
HSPA8	heat shock protein family A member 8
hUC	human umbilical cord-derived
IDO	indoleamine 2,3-dioxygenase
IGF	insulin-like growth factor
IL	interleukin
IP-10	interferon γ-induced protein 10 kDa
iPSCs	induced pluripotent stem cells
LAMP2A	lysosomal-associated membrane protein 2A
LAS	lysosomal autophagy system
LBs	Lewy bodies
l-DOPA	l-3,4-dihydroxyphenylalanine
LGALS	galectin
LIF	leukemia inhibitory factor
LRRK2	leucine-rich repeat kinase 2
MAPK	mitogen-activated protein kinase
MCP-1	monocyte chemoattractant protein-1
MDA	malonaldehyde
miRNA	micro-RNA
MMP-2	metalloproteinase-2
MPP+	1-methyl-4-phenylpyridinium ion
MPTP	1-methyl-4-phenyl-1,2,3,6-tetrahydropyridine
MSCs	mesenchymal stem cells
mTOR	mechanistic target of rapamycin
NCOA4	nuclear receptor coactivator 4
NF-M	neurofilament M
NF-κB	nuclear factor κ B
Ngn	neurogenin
NO	nitric oxide
Nrf2	nuclear factor erythroid-related factor 2
NSE	neuron-specific enolase
NT-3	neurotrophin-3
OCR	oxygen consumption rate
ODC1	ornithine decarboxylase 1
OPG	osteoprotegerin
PD	Parkinson’s disease
PGC-1α	peroxisome proliferator-activated receptor-γ coactivator 1-α
PI3K	phosphoinositide 3-kinase
PINK1	phosphatase and tensin homolog induced novel kinase 1
PKD1	protein kinase D1
PMCA	plasma membrane Ca^2+^ ATPase
PP2A	protein phosphatase 2A
PRKN	Parkin
RANTES	regulated upon activation, normal T cell expressed and secreted
ROS	reactive oxygen species
SA-β-gal	senescence-associated β-galactosidase
SERCA	sarcoplasmic/endoplasmic reticulum Ca^2+^-ATPase
SHEDs	stem cells derived from human exfoliated deciduous teeth
SIRT	sirtuin
SLC7A11	solute carrier family 7 member 11
SN	substantia nigra
SNCA	synuclein alpha
SNpc	substantia nigra pars compacta
SOD	superoxide dismutase
SQSTM	sequestosome
STAT	signal transducer and activator of transcription
TCA	tricarboxylic acid
TFAM	mitochondrial transcription factor A
TGF-β2	transforming growth factor beta 2
TIMP-1	tissue inhibitor of metalloproteinase
TLR-3	toll-like receptor 3
TNF-α	tumor necrosis factor α
TNTs	tunneling nanotubes
UCHL1	ubiquitin C-terminal hydrolase L1
UPS	ubiquitin-proteasome system
VEGF	vascular endothelial growth factor
VSP35	vacuolar protein sorting 35
